# Strengthening Health Security at Ground Border Crossings: Key Components for Improved Emergency Preparedness and Response—A Scoping Review

**DOI:** 10.3390/healthcare12191968

**Published:** 2024-10-02

**Authors:** Diana G. Sami, Sungsoo Chun

**Affiliations:** Institute of Global Health and Human Ecology, The American University in Cairo, New Cairo 11835, Egypt; dianasami@aucegypt.edu

**Keywords:** ground border crossings, emergency preparedness and response, health security, health border management, IHR

## Abstract

**Background:** Ground crossing borders are considered a threat to health security due to their complex and challenging environments. The objective of this scoping review is to identify the main themes crucial for implementing effective health management at ground crossing borders to maintain health security. **Methods:** This scoping study was conducted following the methodological framework developed by Arksey and O’Malley and the Preferred Reporting Items for Systematic reviews and Meta-Analyses extension for Scoping Reviews (PRISMA-ScR). Documents published from January 2005 to December 2023 were searched for using PubMed, Scopus, Web of Science, and UN databases. Two reviewers screened and reviewed eligible studies in three stages: duplicate identification and elimination, title and abstract screening, and full-text assessment. Data were charted and grouped into themes, the frequency of each theme and its percentage was calculated, and then thematic analysis was conducted. **Results:** Forty-five studies met the inclusion criteria. Areas of research were grouped into eight themes: “Infection Prevention and Control measures (IPC) (17%)”, “Collaboration, Coordination, and Partnership (17%)”, “Research and Data Sharing (15%)”, “Build Border Health Capacity (13.5%)”, “Planning (13.5%)”, “Communication (13%)”, “Legislations and Frameworks (7%)”, and “Services and Assistance for At-Risk Groups (4%)”. Through this scoping review, we found that the eight themes are interconnected, and are crucial for implementing effective health management at the ground crossing borders and for better emergency preparedness responses among countries.

## 1. Introduction

In the global health context, a Public Health Emergency of International Concern (PHEIC) refers to any sudden or evolving situation that poses a significant threat to public health, often overwhelming local or national capacities and requiring a coordinated international response [1]. These emergencies can arise from natural disasters (e.g., earthquakes, floods), infectious disease outbreaks, man-made crises (e.g., armed conflicts, or chemical, nuclear, or biological accidents), or due to climate change [2]. These disasters can also trigger forced migration, adding to the burden on global health security [3].

The world is facing significant health challenges arising from infectious disease outbreaks, conflicts, and natural and environmental disasters, leading to large numbers of displacements and humanitarian crises. Examples of infectious diseases that are considered health emergencies include COVID-19, Ebola, cholera, Dengue fever, Monkey Pox (MPox), Avian Influenza A (H7N9), Influenza A (H1N1) virus, Zika virus disease outbreak, and Middle East Respiratory Syndrome (MERS-CoV) outbreak [4]. Health emergencies due to natural disasters include the Pakistan crisis caused by flooding in 2020, earthquakes in Turkey and the Syrian Arab Republic in 2023, and Tropical Storm Daniel in 2023 which caused deadly flooding in Libya [5,6,7]. Drought and food insecurity are observed in the greater Horn of Africa [8]. Additionally, the world is experiencing an increase in conflict and civil unrest in countries such as Iraq, Syria, Libya, Afghanistan, Ukraine, Somalia, and Yemen. Violent clashes in Sudan on 15 April 2023 forced over 8.6 million people, including refugees, internally displaced people (IDPs), and asylum seekers, to flee their homes. Furthermore, civilian deaths and injuries have occurred as a result of the ongoing conflict between the Israeli military and Hamas in the occupied Palestinian territory, which began on 7 October 2023 [4]. These disasters disrupt health systems and pose significant public health threats by increasing the risk of spreading water- and vector-borne diseases, diarrheal outbreaks, malaria, and many other threats.

Disease knows no borders because disease pathogens can be transmitted across geographical borders through the movements of humans, animals, and goods [9]. Ground crossing borders (GCBs) are significantly impacted by migration flows resulting from political instability and climate change [10]. GCBs are known for their more complex environments compared to other points of entry (POEs) such as airports and seaports. Land borders are considered potential vulnerabilities in global health security, presenting greater challenges in implementing the International Health Regulations 2005 (IHR 2005) due to inadequate infrastructure, understaffed and under-resourced official borders, and a lack of robust shareable surveillance systems [11].

GCBs have limited healthcare facilities, limited financial resources, inadequate health staff, and limited training on standard operating procedures (SOPs) [12]. Some countries lack knowledge of the IHR 2005 including designated sites, and reporting procedures among border officials (BOs) and healthcare practitioners (HPs) [10]. They may not understand concepts such as asylum seekers, irregular migration, displaced persons, screening, and human trafficking. There is no specialized training on the health aspects of migration and human trafficking, vaccination, infection, modes of transmission, and occupational health [10]. All of these limited resources hinder the implementation of surveillance activities at POEs [13]. Although regulations for handling health-related emergencies may be available in some countries, preparedness exercises may not be conducted [13]. Guidelines for maintaining quarantine during lockdowns are lacking, there is inadequate personal protective equipment, some POEs do not have running water for handwashing, and there are inadequate waste disposal facilities [13]. There is an absence of community involvement and effective risk communication, inadequate case management at POEs, and insufficient isolation rooms. Some POEs lack isolation centers, screening rooms, and workstations. Additionally, there is a lack of quarantine facilities for imported animals posing a potential risk to public health [14]. Furthermore, there is a shortage of specialized psychological care, a limited presence of non-governmental organizations (NGOs), and a lack of specialized treatment and protection programs for victims of torture and violence in certain countries [13].

Furthermore, the evaluation of migrants’ health conditions and the provision of support appropriate to their needs are hindered by the lack of evidence-based research, the unavailability of standardized medical screening for irregular migrants, and the absence of statistics about migrant health. These factors also increase the risks associated with the border population and staff, especially in the event of an epidemiological outbreak. Providing health services to migrants also presents challenges for health professionals. These challenges include communication issues, addressing prejudices and cultural differences, and working with individuals from diverse cultural and risk backgrounds. Additionally, health professionals must be sensitive to the physical and mental health needs of vulnerable populations, such as trafficking victims, smuggled migrants, and minors [13].

The lack of coordination among border officials and their counterparts in national and local public health services leads to a lack of reliable information regarding the cross-border epidemiological situation [10].

Furthermore, there is a significant lack of knowledge and information regarding the duties and organizational structure of the corresponding institutions in the neighboring country [10]. Conflicts of interest, along with governance complexity, can act as barriers to effective cross-border resilience [10]. These gaps raise concerns about the readiness of the authorities in the land border region (health and police/guards) in the event of a crisis [13].

Global health security is a crucial component of pandemic preparedness strategies, with GCB efforts playing a central role. GCBs are essential in controlling the spread of infectious diseases such as COVID-19, Ebola, malaria, and other zoonotic diseases that can easily cross borders. Effective vaccination campaigns can prevent the transmission of vaccine-preventable diseases like measles, polio, and yellow fever. Additionally, GCBs can serve as pathways for transboundary pollutants and the spread of drug-resistant bacteria posing significant public health threats.

Due to increasing conflicts and wars globally, biosecurity and bioterrorism have become threats that require effective health management at GCBs to detect and contain them before they spread. Political instability and wars have forced many people to flee their countries, with these individuals often lacking access to adequate healthcare, highlighting the need for coordinated strategies across borders. Furthermore, GCB strategies must address human rights and health issues related to trafficking, as vulnerable populations on the move are at higher risk of exploitation facilitated by porous borders.

Given the porous nature of GCBs and the health threats they pose, this scoping review aims to provide information to inform policy on key themes essential for effective cross-border management. These themes include maintaining global health security, preventing the spread of disease, ensuring access to health services for refugees and migrants, addressing environmental and zoonotic threats, protecting human rights, and establishing laws to prevent crimes and counter-trafficking.

## 2. Materials and Methods

This scoping study was conducted following the methodological framework developed by Arksey and O’Malley for scoping review studies [15], and the Preferred Reporting Items for Systematic Reviews and Meta-Analyses extension for Scoping Reviews (PRISMA-ScR) [16,17]. These guidelines were followed to ensure consistency in the findings and to enhance the usefulness of collective knowledge in policy-making decisions. By using both approaches, a complementary and comprehensive process is established. The Arksey and O’Malley framework aids in identifying the scope and provides the methodological structure for conducting a scoping review, while PRISMA-ScR ensures standardized reporting of the process, ensuring that each step is thoroughly documented and presented in a structured manner. Together, they guarantee clear reporting.

### 2.1. Research Questions and Objective

Research Question: What are the best practices for effectively managing health at ground crossings during emergencies?

We hypothesize that effective health border management at ground crossings during emergencies requires an integrated and multidisciplinary approach, involving engagement from various stakeholders at both national and international levels. This collaboration is essential for improving preparedness and response efforts.

The objective of the scoping review is to identify the main themes crucial for implementing effective health management at ground crossing borders to maintain health security.

### 2.2. Relevant Studies Identification

Electronic databases were searched to identify relevant data from published and grey literature. The databases used for the search included PubMed, Scopus, Web of Science, and UN databases (World Health Organization and International Organization for Migration). Keywords used for the search were International Health Regulation, health security, land border, ground border, and crossing border. Boolean operators were used as conjunctions to combine these keywords in the search for focused results. The research term used was as follows: “(((International Health Regulation) OR (health security)) AND ((land border) OR (ground border) OR (crossing border)))”.

### 2.3. Eligibility and Selection of Studies

The identified studies had to meet the following inclusion criteria to be included:

1. Written in English.
2. Published between January 2005 and December 2023, as the IHR was first announced in 2005.
3. Given that health border management involves numerous stakeholders, the types of papers included were not restricted to peer-reviewed articles to gather as many opinions as possible. It was important to incorporate reports from organization websites and grey literature to map the data.

We excluded papers that were scoping or systematic reviews, written in languages other than English, not open access, and not focused on ground crossing borders.

All papers resulting from the search were imported and duplicates were removed using the Zotero citation machine. Two reviewers initially evaluated the abstracts and titles of the papers to exclude those that were not relevant, that were scoping or systematic reviews, or were not written in English. The same two reviewers then conducted full-text screening, excluding articles that were not open access and did not focus on ground crossing borders. Color coding was used to differentiate between the included and excluded studies. Regular meetings and discussions were held to resolve any discrepancies and reach agreement. The study selection process was documented using a PRISMA flow diagram illustrating the number of studies identified, screened, excluded, and included in the review.

### 2.4. Charting of Data

Two reviewers used a standardized data table for the papers included in the study. Each paper was assigned a number, and the table included a header with the year of publication, publication type, publisher, location, main category, and a column for a summary of the paper. The reviewers thoroughly screened each paper to summarize and identify the main themes. The data from the table were then cross-checked to confirm the grouping of the themes. Discussions were conducted to harmonize the themes and resolve any discrepancies.

### 2.5. Collating, Summarizing, and Reporting Results

The papers were categorized according to the sections in our data charting table, with the results considered relevant to the goal of our scoping study, displaying the variety of research found. The frequency of each theme and its percentage was calculated. The data were then subjected to thematic analysis to map the current literature.

## 3. Results

### 3.1. Overall Results

**Figure 1:**
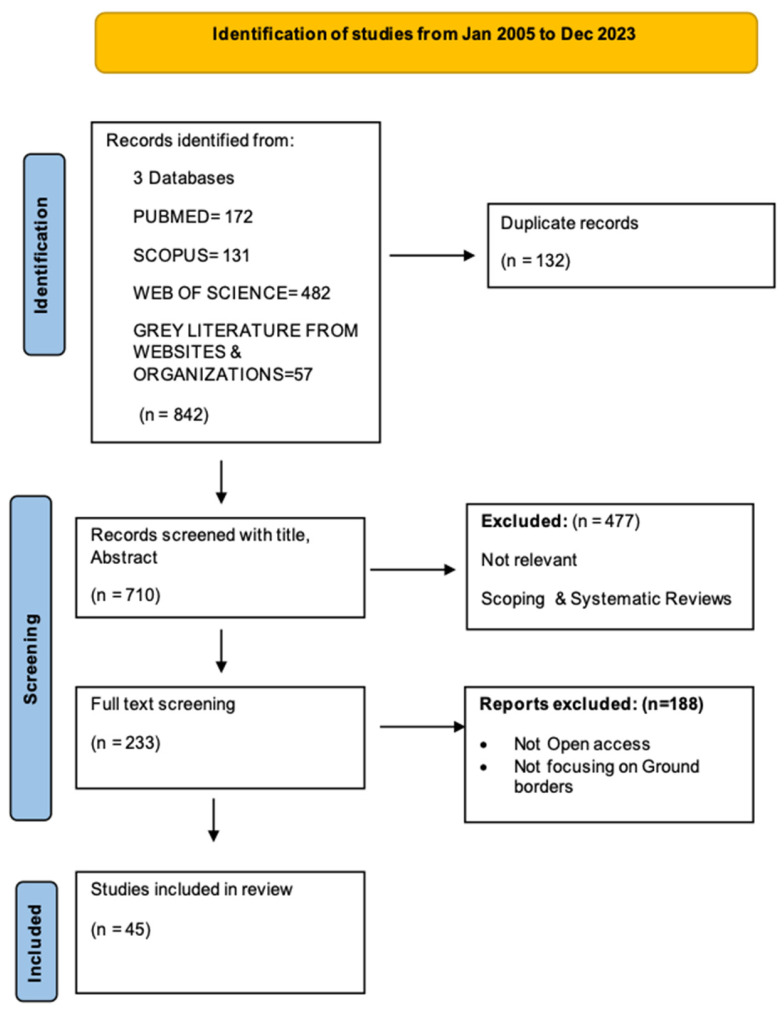
PRISMA flow diagram illustrating the article search and selection process.

Initially, 842 articles were retrieved from three databases along with grey literature from UN websites and organizations. After removing 132 duplicates, 710 articles remained. Following title and abstract screening, 477 articles were excluded for being irrelevant, systematic or scoping reviews, or not written in English, leaving 234 for full-text review. Then, 188 articles were excluded again as they were not freely accessible and did not focus on ground borders. This resulted in 45 papers meeting the inclusion criteria, focusing on the challenges and key factors needed for effective health border management at ground borders (Figure 1).

Full-text screening was conducted for the 45 papers and each paper was tagged with relevant key terms. This process led to the grouping of papers into eight main themes for effective health border management at the ground borders. The eight themes are as follows: (I) Infection Prevention and Control measures (IPC) (17%); (II) Collaboration, Coordination, and Partnership (17%); (III) Research and Data Sharing (15%); (IV) Building Border Health Capacity (13.5%); (V) Planning (13.5%); (VI) Communication (13%); (VII) Legislations and Frameworks (7%); and (VIII) Services and Assistance for At-Risk Groups (4%) (Table 1).

The full characteristics of the extracted studies are summarized in Table S1.

Our results support the idea that health border management at ground crossings during emergencies is inherently multidisciplinary. This is due to its complex interplay with multiple stakeholders in various fields (governmental and non-governmental) who collaborate at both national and international levels to ensure both security and public health.

### 3.2. Identified Themes Should Be Included for Effective Health Border Management

#### 3.2.1. Infection Prevention and Control Measures (IPC)

Land border Quarantine Stations (QSs) have unique characteristics. Because there is less control governing the transportation of goods across these borders, land borders are more susceptible to dangers and health issues. To address these threats, the QSs serving such borders should shift from providing inspection to providing strategic public health leadership. QSs must take on special roles and partnerships with other partners, such as community-based organizations, to attain strategic leadership and support emergency preparedness planning, public health research, and prevention initiatives among foreign travelers and migrants [18].

For QSs at land borders, a variety of actions are required. These include cross-border surveillance, preparedness, creating SOPs for reporting illnesses at land crossings among travelers, and working and communicating often with a variety of partners to enable cross-border travel, binational communication, and the transfer of public health supplies. This is essential to ensure continued collaboration [18].

##### Screening, and Referral and Case Management

The Ebola virus disease (EVD) outbreaks during 2014–2016 in West Africa have placed pressure on health systems, making it necessary to manage suspected cases at POEs to prevent the disease from spreading across borders. It is important to follow risk evaluations and WHO standards for managing EVD, while also considering the specific characteristics of each POE [19]. According to the IHR, designated PoEs should have SOPs and emergency plans in place for public health emergencies. These plans should outline screening procedures, health forms, business continuity plans, and transportation facilities for sick travelers, and health referral systems should all be mentioned in SOPs and guidelines [12].

The WHO has provided guidelines to POEs for designing entrance and exit screening. The guidelines cover data management, medical referral, communication strategies, resource requirements, screening methodology, tools, and the legal issues related to screening [20,21].

During the 2014-2016 EVD epidemic in West Africa, the Centers for Disease Control and Prevention (CDC) implemented travel and border health precautions to prevent the international spread of the disease, protect and educate travelers and communities, and minimize disruptions to international trade and travel.

A study conducted between 20 April 2009 and 31 July 2009 evaluated the public health actions taken at international POEs in response to the pandemic (H1N1) 2009 virus. The results showed that implementing policies aimed to prevent the entry and spread of infectious diseases that impact international travel and trade. The effectiveness of screening procedures is influenced by resources such as trained workers, technology, and quarantine procedures. The study also suggests establishing an international network of public health partners to facilitate communication about health-related concerns [22].

##### Surveillance, and Early Warning Alert and Response

To promptly identify public health incidents, coordinate and exchange epidemiological data, and implement suitable public health interventions for illness prevention and control, GCBs should establish efficient surveillance systems [12].

Maintaining sanitary standards, and capacity building for vector surveillance and control programs, are crucial for GCBs. This is achieved by providing technical guidance on the optimal use of resources, planning, monitoring, and decision-making [23]. To implement the Early Warning and Response System (EWARS) and respond to all acute health events, the IHR broadens the typical infectious disease notification. This is done in response to the rapidly changing environment, which includes factors such as increased international trade and migration, the emergence of new pathogens, environmental disasters, accidents involving chemicals and nuclear materials, and bioterrorism. Financial, material, and human resources are needed for the installation and enhancement of the EWARS. Planning is necessary to ensure the effective use of resources in the context of limited health resources. To reduce the detrimental effects on the affected population’s health, the EWARS seeks to improve the sensitivity of detection, the degree of risk assessment, and the promptness and efficacy of the response to acute public health hazards. This can only be accomplished by addressing all local priority health concerns, potential national sources of emergence, and additional disease activity indicators through the use of detection technologies, data management protocols, and information-sharing processes; these are collectively known as epidemic intelligence. Integration of systems for acute public health risk preparedness and response is also necessary for the prompt execution of effective control measures [24].

The One Health approach aims to prevent the spread of cross-border zoonotic diseases. Uganda’s borders are highly porous to animal movement, which may contribute to zoonotic diseases. Therefore, proactive measures are taken to prevent cross-border and inter-district spread of zoonotic diseases. A toolkit similar to the population mobility mapping tools was modified and tested to gather information on animal movements and interactions to inform One Health initiatives. The Piloted Animal Population Connectivity Across Borders (PopCAB) can gather a wide range of detailed information to describe the movement of animals. To create zoonotic disease prevention, control, and emergency plans at the national and international levels, information on animal migrations and interactions is essential. The application of an animal movement mapping exercise emphasizes the significance of cross-sectoral collaboration to advance One Health border health strategies [25].

##### Vaccination

The application of public health measures such as mass vaccination campaigns for vaccine-preventable diseases (VPDs) should be coordinated internationally and integrated into emergency plans to optimize their effectiveness. A review of relevant health documents such as vaccination certificates should be available to minimize the risk [11].

#### 3.2.2. Collaboration, Coordination, and Partnership

Several expert meetings have highlighted a lack of coordination between the National Public Health Surveillance System (NPHSS) and authorities at PoEs who receive or generate information on health incidents related to travelers, products, and conveyances. By enhancing coordination and communication between PoEs, the NPHSS, and authorities, IHR 2005 can be implemented to strengthen national capacities for the prevention, detection, and control of events [26].

A case study demonstrated the impact of collaboration and communication in containing Lassa fever in West African countries. Lassa fever is a significant public health concern due to its rapid spread over land borders and a 70% death rate without treatment. The case report indicated that the Guinean Ministry of Health (MoH) declared an outbreak after a patient from forested Guinea tested positive for LASV through reverse transcriptase–polymerase chain reaction (RT-PCR) in Liberia. The study emphasized the importance of active case finding, contact tracing, laboratory support, and risk communication for disease detection in the community and for healthcare worker training.

The identification of transmission sources and patterns relies on data sequencing. Research has shown that through communication and cross-border cooperation within the framework of Integrated Diseases Surveillance and Response (IDSR) and IHR, such risks can be swiftly controlled. The study underscored the necessity of integrating private healthcare facilities into IDSR by providing facility materials, training, and routine surveillance data collection for healthcare professionals. Within 24 h of notification, Guinea successfully conducted a preliminary epidemiological examination through the implementation of IDSR [27].

Another example illustrating the significance of coordination is the development of the Caribbean Region Global Health Security Agenda (GHSA) Roadmap to enhance health security in the Caribbean region. Uncontrolled disease outbreaks pose a significant threat to the Caribbean due to its small health systems and reliance on tourism. The Caribbean region faces diversity and susceptibility to external shocks, whether natural or man-made. As a proactive measure to bolster health security, a regional self-assessment was carried out from September 2016 to May 2017, resulting in the development of the GHSA Roadmap. The creation, approval, and implementation of the roadmap in the Caribbean region through improved multisectoral coordination, cooperation, and communication is considered a significant milestone and an innovative approach for other regions to consider and adopt [28].

Establishing new and existing partnerships was crucial in containing the Ebola epidemic in West Africa from 2014 to 2016, including collaborations with non-governmental organizations, the United States Department of Homeland Security, health authorities, and global organizations such as the WHO and the IOM [29].

The partnership approach and the programs’ related effects on the detection, reporting, and response to epidemic-prone diseases in Guinea offer valuable insights for developing surveillance infrastructure and capability in comparable contexts. Through collaboration with key laboratory stakeholders and One Health, this integrated and cooperative strategy supports prompt, accurate, and comprehensive data transfer from the community to the national level. It has also contributed to the establishment of significant cross-sector linkages. Guinea has made significant progress towards meeting the surveillance and reporting targets outlined in the Global Health Security Agenda (GHSA) and the IHR due to the technical expertise of the surveillance partners and their close coordination with the MoH and the National Health Security Agency [30].

Comparative policy analysis examines the responses of Association of Southeast Asian Nations (ASEAN) member countries to the COVID-19 pandemic, focusing on the limits of regional cooperation and the potential for coordinated efforts. Cooperative frameworks within ASEAN could aid in formulating collective responses to stop the spread of COVID-19 infections. Current frameworks for health security cooperation could serve as a foundation for more coordinated regional efforts, organized around the concept of “inclusive regionalism”, to address collective challenges by fostering cooperation and collaboration among member countries [31].

Inclusive regionalism promotes coordinated efforts during pandemics by facilitating communication exchanges, information sharing, and collective action among member states to prevent and eliminate the spread of subsequent waves of the virus. By fostering collaboration and cooperation, inclusive regionalism enables the provision of financial and technical assistance through the establishment of the ASEAN COVID-19 Response Fund. This fund assists member states lacking adequate health facilities, services, and expertise, aiming to promote solidarity and mutual support among ASEAN member states to effectively respond to the crisis and mitigate its impacts on health, economy, and society [31].

Crisis management in modern times is a complex task that requires a high degree of coordination among many participants. In cross-border regions, coordination involves coordinating emergency responses through collaborative capacity planning (technical and manpower planning) to avoid overwhelming responders with the complexity of cross-border coordination [10].

During a state of emergency, there is civil–military collaboration. For example, the Territorial Defense Force (TDF) in Poland performs crisis management tasks, such as search and rescue operations, evacuation, and property protection, and cooperates with local communities, during disasters. They are also involved in disaster planning and response, particularly in providing medical support and aeromedical assets during natural disasters and mass gatherings [32].

Regional coordination can enhance pandemic response effectiveness by promoting harmonization of travel rules, facilitating cross-border trade and transport, and aligning testing protocols among member states. By working together to standardize risk assessment, thereby enhancing data consistency and availability, interoperability of digital health credentials, and response, regions can improve communication and cooperation, leading to a more unified and effective approach to pandemic-related measures. Additionally, regional coordination can help address challenges such as limited vaccine access, capacity constraints, and the politicization of lockdowns and travel restrictions, ultimately contributing to a more efficient and coordinated response to public health crises [33,34].

Multisectoral engagement is necessary and should include all pertinent ministries, such as livestock, customs, health, UN agencies, and NGOs. All levels of government must assist these agencies, both in terms of institutional necessity and evidence [12].

At GCBs, all border agencies should actively participate in integrated coordination procedures.

Establishing and identifying the essential components for cross-border coordination and information exchange is necessary. Public health incidents, possible security concerns that might affect the response to an outbreak, information exchanged on mass movements, peak hours, travel restrictions, and preparedness and response activities should be reported to the IHR focal person [12].

Enhancing and establishing cooperation between border police/guards and national public health agencies, as well as with partners on the other side of the border, is vital. For example, some UN agencies collaborate with authorities to strengthen national and regional contingency plans, provide technical assistance to ensure the safety and health of people on the move, and engage in coordination and partnerships with national and regional authorities, and with relevant health authorities, to facilitate access to emergency healthcare for migrants [35].

Following the Ebola outbreak in Congo in 2018, different UN agencies such as the IOM and WHO worked closely with the Congolese MoH to contain the outbreak and address the needs of the affected communities. Activities included population mobility mapping, risk communication, infection prevention and control, strengthening POE surveillance through the deployment of surveillance staff, and daily flow monitoring to improve response [36].

#### 3.2.3. Research and Data Sharing

Research and data sharing are crucial for preventing cross-border health threats. Research generates evidence that guides planning for future public health emergencies. Investigating ways to improve communication on public health-related activities at POEs is critical to promoting cross-border cooperation, timely and consistent exchange of expertise, and the sharing of lessons learned [22]. Novel techniques and tools that strengthen the evidence base to develop more effective intervention and monitoring strategies are essential given the threat to national and international financing [37]. Further research and piloting of developed materials are needed to establish a common toolkit for migration and health. Standardized data collection plays a crucial role in policy evaluation by providing a reliable foundation for assessing the impact of interventions or programs on public health and migration issues. Additionally, standardized data collection methods enable better comparability of data across different countries and institutions, facilitating more effective analysis and evaluation of policy outcomes [34,38]. Assessments and situational analysis are crucial for capacity development in terms of personnel deployment, training, SOP implementation, contingency planning, and coordination and communication mechanisms [12].

Population Mobility Mapping (PMM) is an important tool widely used to understand human mobility dynamics and identify locations vulnerable to public health risks. It aims to inform public health interventions that prevent, detect, and respond to public health emergencies and international health threats along the mobility continuum.

The objectives of PMM include characterizing migrants’ profiles and major mobility routes in a specific geographical area. It also aims to identify priority locations and vulnerable areas susceptible to infectious disease outbreaks and other public health concerns with limited resources for response and preparation. Additionally, it aims to improve emergency preparedness and response capabilities by allocating resources and designing public health interventions and plans at designated priority locations.

PMM consists of three linked components that inform emergency preparedness and response for public health: (I) Participatory Mapping Exercises involving facilitated group discussions with key informants to share knowledge about mobility and vulnerable spaces in a specific area through illustrations on a base map; (II) Field visits to priority sites identified during the participatory mapping exercise to confirm and gather additional information through direct observations and key informant interviews on-site; and (III) Flow Monitoring, which includes surveys to collect data on population flows and mobility characteristics at POEs or locations with high mobility.

Not all PMM components can be implemented at all times, so the appropriate components should be chosen in consultation with relevant authorities and partners. This tool is used for public health preparedness and response in many countries in various contexts, including developing mobility-centered approaches for routine immunizations in children, and in response to events like the COVID-19 pandemic, EVD, and plague [39].

Planning and executing field simulation exercises (FSXs) require a significant amount of time, money, and organizational expertise [40]. Despite these challenges, FSXs provide an opportunity to assess all operational capabilities, including cross-sector coordination. The Republic of Kenya and the United Republic of Tanzania conducted a cross-border FSX at the Namanga One Stop Border Post from 11 June to 14 June 2019, as reported by the East African Community Secretariat. The exercise was planned and coordinated by the Department of Health Security Preparedness at the World Health Organization, which served as the technical lead. The goal of the FSX was to assess and enhance multisectoral epidemic preparedness and response in the East Africa region. It provided an opportunity for individual learning, and for the enhancement of national emergency management and response coordination capabilities [40].

Countries must allocate resources and improve their capacity for preparedness and response. It is recommended that countries regularly undertake FSXs to prevent outbreaks of diseases that can jeopardize public health, economic stability, and the lives and livelihoods of people in their countries and regions, as observed during the COVID-19 pandemic [40].

Any National Health Plan should place a high premium on increasing surveillance. To increase IDSR, thereby enhancing surveillance at POEs, developing CBS, and facilitating real-time surveillance and reporting, the US CDC and its partners offer technical support for the development of complementary instruments and processes. Strong ties with the Ministry of Health and participation at all levels of the surveillance system have made this achievable [40].

Project-based research is essential for enhancing resilience. To improve the methodological assessment of cross-border areas, a deep understanding of the diversity and complexity of disaster risks in these regions, their consequences, and the available capacities is required [10].

Benin, Nigeria, and Togo, along with collaborating organizations, made strong efforts to enhance cross-border public health coordination in response to Lassa fever outbreaks. Following three outbreaks between 2017 and 2019, they improved their binational and multinational communication and coordination to quickly identify and address at-risk cases and communities. This was achieved through the implementation of procedures for public health collaboration through meetings and fieldwork to characterize population movement patterns. Benin showed an improved ability to gather and distribute comprehensive cross-border travel records and related risk assessments during the outbreak, utilizing the recent PopCAB for better surveillance. These efforts led to enhancements in information collection and sharing, coordination, and collaborative efforts to reduce the international spread of diseases. Coordinating public health information on a multinational scale in the context of high-volume cross-border population migration and community connectivity is challenging. Adapting public health systems to consider unofficial migration routes, cross-border travel trends, and cultural elements affecting relocation is crucial [41].

Quantifying cross-border movements was vital for strategic intervention planning and collaboration at national and regional levels to enhance malaria control efforts in East Africa [37]. National census data were utilized to analyze and model cross-border migration patterns, identifying hotspots of origin-specific immigrants from neighboring countries in Kenya, Tanzania, and Uganda [37]. The results showed fluctuating immigration patterns between countries of origin and destination, leading to variations in the propensity to import malaria and differences in migration processes and transmission risks [37].

#### 3.2.4. Build Border Health Capacity

The Joint External Evaluation (JEE) tool was designed to assess a nation’s ability to maintain health security under the IHR and to provide recommendations across various technical areas reviewed by experts. Through the JEE, nations can prioritize opportunities for improved preparedness, response, and action, identify critical requirements within their health security system, and communicate with partners and donors. The first edition of the JEE was released in February 2016. In April 2017, the WHO convened a global meeting with over 90 technical experts from around the world to discuss enhancements and recommendations for the JEE. These recommendations were incorporated into the second edition of the tool. The JEE consists of 49 indicators across 19 technical areas grouped into four core areas: prevention, detection, response, and IHR-related hazards and points of entry. To prevent and reduce the likelihood of outbreaks and other public health events as defined by the IHR, the JEE considers early threat detection as crucial for saving lives, and emphasizes the importance of multisectoral coordination and communication, both nationally and internationally, for effective response.

Additionally, it is important to evaluate IHR capacities at points of entry, during chemical events, and in radiation emergencies. JEEs are characterized by voluntary country participation, a multisectoral approach by both external teams and host countries, transparency and openness in data and information sharing, and the public release of reports [42].

##### Training

Building capacity and providing personnel training at ground crossings should be a top focus for emergency response and preparedness programs [12]. The BO’s role plays a significant part in preventing the spread of the virus, while also emphasizing the need to protect their own health. The International Organization for Migration (IOM) supports migrants and border authorities through various tools and training programs. They provide assistance by developing SOPs for BOs, health training curricula, and communication tools to enhance communication with migrants [43]. They have created training materials to reduce threats to public health, enhance the capabilities of public health personnel and border management, and facilitate proper medical care for migrants as a basic human right. Recommendations for public health services and detention procedures in designated border sectors are also included. Topics including intercultural competence, mediation, and communication skills are covered as well [43,44]. Psychological support for staff working with migrants is urgently needed, along with training on human rights, safety, and security in the workplace [45].

In addition to receiving training on how to interact with vulnerable populations, it is essential to understand global migration patterns, be aware of the implications of migration for public health, and be sensitive to the physical and mental health needs of vulnerable groups, such as victims of trafficking, smuggled migrants, and minors [45].

##### Diversity of Workforce

Recommendations include strengthening GCBs’ health security capacities through multi-sector engagement, and increasing and diversifying health personnel. Diversity in the workforce is crucial during cross-border emergency operations because it brings together a range of skills, perspectives, and cultural insights that enhance problem-solving and decision-making. In emergencies, especially those involving different countries, the ability to understand and communicate across language and cultural barriers is essential. A diverse team can bridge gaps in local knowledge, customs, and practices, ensuring that aid and interventions are appropriate and sensitive to the affected populations. It also fosters inclusivity, allowing for a broader understanding of the challenges faced by various groups, leading to more effective and equitable responses. Diversity strengthens teamwork, resilience, and adaptability in high-pressure situations where quick, coordinated action and improving information collection and analysis systems to align with IHR 2005 guidelines are essential [12]. Examples of workforce teams that should be available at GCBs include medical professionals, logistics and supply chain personnel, humanitarian aid workers, security personnel or BOs, government immigration and legal advisors, communications and IT specialists, public health and Water, Hand, Sanitation and Hygiene (WASH) experts, and psychosocial support workers.

##### Facilities, Infrastructure, and Supplies

A proper structural infrastructure is essential for screening, quarantining, and physical distancing. Additionally, a well-established infrastructure makes it easier to provide other necessities such as drainage systems, WASH stations, electricity, connectivity, equipped labs, appropriate detention centers, enough PPE, hospitals, and information and communication technologies [12]. Conditions and procedures in detention should fully respect migrants’ dignity and fundamental rights. The distribution of hygienic supplies should be secured, providing a balanced nutritious diet. Migrants in closed centers should have access to fresh air. Detention centers should establish agreements with health services to provide healthcare to irregular migrants, vulnerable migrant groups, asylum seekers, and foreigners granted subsidiary protection. They should also offer psychosocial support to asylum seekers who have been exposed to torture, rape, or other forms of violence [45].

##### Financial Resources

For both developed and developing countries, providing financial assistance for capacity building for effective border management can be challenging. It will be necessary to prioritize the level of cross-border cooperation initiatives within the limitations of current funding sources. Securing funding to implement these collaborative agreements may be facilitated by involving a diverse group of stakeholders, including government ministries, traditional community leaders, non-governmental organizations, business executives, and other members of civil society [11]. Participating countries should establish a cooperative approach to raising funds to support the designated activities in each country [11].

#### 3.2.5. Planning

Following the IHR, designated PoEs should have SOPs and emergency plans for public health. The goals of the plans should be to prevent the spread of disease internationally while allowing PoE authorities to avoid unnecessary delays and restrictions, identify potentially affected individuals as soon as possible, and implement WHO recommendations for controlling outbreaks. To achieve this, POEs must have resources, space, and a sufficient number of trained personnel for health assessments. There should also be prompt and effective communication between the national health monitoring system and PoE health authorities, as well as between officials in the transport and public health sectors. Any suspected cases must be reported to PoE health authorities as soon as possible [19].

It is recommended that specific SOPs be created and distributed to frontline BOs at ground crossing borders to provide guidelines for administration, detection, notification, IPC measures, infrastructure considerations, and traveler processing tailored to PHEIC [46]. WASH services should be implemented in all IPC programs. This can be achieved by supplying clean drinking water, improving drainage systems, ensuring the availability of restrooms and washrooms, installing appropriate waste disposal systems, providing training on waste management, through disinfection, and through PPE use [12]. Procedures for cross-border coordination and communication between countries should be created and information should be shared both nationally and internationally [12].

PoEs should have an effective and acceptable system for record-keeping, documentation of vehicle inspections, and public health measures applied. Access to resources needed for health assessments, rapid risk assessments, and the implementation of interim control measures must always be available [26].

A Public Health Emergency Contingency Plan (PHECP) should be created and kept up to date at designated POEs following the IHR 2005. The WHO has developed guidelines that offer the National Points of Entry Health Authorities (NaPHAs) a suggested methodology, framework, and rationale for creating a PHECP at designated POEs. It also highlights key requirements of the IHR 2005 specific to designated POEs that can be included in the plans. It is crucial to align POE plans with the national public health and emergency response framework [47].

The CDC assists countries in developing and strengthening their National Action Plan for Health Security (NAPHS) following the JEE. Developing a NAPHS helps countries identify activities that align with JEE technical areas and prioritize them for implementation. The resulting plan details activities necessary to close any gaps in a country’s health security capacity. The CDC collaborates with partners to promote coordination, cooperation, communication, setting priorities, organizing resources, implementing projects, and monitoring progress [48].

Since the declaration of the 10th EVD outbreak in the Democratic Republic of the Congo (DRC) on 1 August 2018, several neighboring countries have been implementing preparedness efforts to prevent the spread of EVD across international borders. Key activities in EVD preparedness efforts include developing national EVD contingency plans, engaging with multiple government sectors and agencies, establishing Incident Management Teams, enhancing surveillance systems, developing risk communication messages, reinforcing screening and infection prevention measures, constructing EVD isolation units, coordinating with stakeholders, cross-border collaboration, and conducting large-scale simulation exercises to evaluate operational capabilities. The country’s EVD readiness has improved, allowing for quicker identification, investigation, and response to EVD alerts due to ongoing financial and technical support from partners [49].

The Uganda Ministry of Health created EVD preparedness plans tailored to its unique multisectoral public health system and complex community ties with the DRC through border health readiness evaluations and risk mapping. Through preparedness assessments and risk mapping, gaps were identified, leading to a tailored plan that includes actions to improve EVD surveillance, laboratories’ and healthcare professionals’ capacities, supply provision to priority areas, strategic building of treatment facilities, and to enhance EVD risk communication [50].

Building sustainable IHR capacities at POEs and along border regions is a goal of the CDC International Border Team, which collaborates with the governments of Nigeria, Togo, and Benin, as well as Pro-Health International (PHI) and the Abidjan–Lagos Corridor Organization (ALCO). They have achieved notable success in implementing low-resource initiatives to increase border health capacities. Implementing a comprehensive border health strategy involving local, national, and regional sectors is the best way to secure border health security for resource-limited countries with porous land borders and high levels of cross-border mobility. Strengthening partnerships to enhance cross-border and multisectoral collaboration and communication is essential to improving global health security [51].

#### 3.2.6. Communication

Communication and community engagement are crucial elements for IPC at ground crossing borders. Community engagement has been shown to have impacts beyond community-based surveillance (CBS) in Guinea. The participation of communities in border regions can positively affect initiatives to enhance monitoring and other health security capabilities. Selecting border health agents from these communities can improve understanding, cooperation, and communication, increasing the likelihood of promptly detecting potential health security concerns at borders [30].

Effective communication and information sharing are vital for both crisis management during disasters and pre-crisis preparedness. Sharing communication patterns, establishing contacts with individuals, having mutual knowledge about emergency procedures in partner regions, and maintaining the flow of information are essential for effective border management. Social media, particularly in cross-border scenarios, has served as a boundary object in recent years, contributing to common communication patterns and fundamental knowledge within crisis response networks. This has helped to reduce language barriers and promote cooperation. However, occasionally, social media has also been shown to exacerbate confusion. Therefore, for better utilization of communication technology, organizations and individuals involved in cross-border management critically require insights and training [10].

Policymakers are encouraged to develop effective public health policies that ensure health education messages are communicated early to travelers and drivers: for example, providing health education at landing sites, tailoring messages to the target audience, and selecting trustworthy message conveyors and communication channels, such as local authorities or medical professionals, to enhance message credibility [52].

To effectively address patients’ health issues, healthcare personnel must be culturally competent. Understanding and respecting patients’ cultural beliefs, values, and behaviors fosters better patient outcomes through improved communication and trust [13].

The IOM created a translation tool called “The Migration Translation Application” (MiTA) to facilitate communication between border and migration management authorities and migrants. This tool enables communication with migrants through a set of pre-defined and pre-recorded questions in different languages [43]. Additionally, they developed “The Support for Migrants Application”, providing migrants in the Western Balkans with a reliable source of information and guidance. This includes the latest advice on government, UN, and non-governmental organization services in the area, such as housing, legal assistance, medical support, specialized organizations for women and children, and aid for victims of crimes like human trafficking [53].

#### 3.2.7. Legislations and Frameworks

**The International Health Regulations 2005 (IHR 2005)** represents a legally binding commitment by 196 countries to enhance their ability to identify and report potential global public health emergencies. The IHR requires all countries to be capable of identifying, evaluating, documenting, and managing public health incidents [48,54]. It assists States Parties in assessing their current capacities and determining the necessary capabilities at POEs, such as airports, ports, and ground crossings [54]. The IHR also allows for the assessment of the implementation of core capacity requirements, identification of existing capacities, and establishment of compliance measures for each requirement. It aids in planning for the improvement, expansion, and maintenance of these core capacities. The updated version of the IHR includes analytical tools to evaluate potential international public health risks of international concern, case definitions of diseases, PHEIC, and clearer definitions of public hazards. It covers biological, chemical, and radiological threats implicitly.

Moreover, the updated IHR 2005 requires countries to be accountable for reporting significant incidents, which is closely linked to their ability to determine the cause of an event. Therefore, the IHR 2005 also calls for enhanced national capacity for prevention, and preparedness and response, as well as for global public health emergencies, global partnerships and collaboration, rights, obligations, accountability, and monitoring [54,55].

**Trade regulations** provide measures to protect goods and individuals from the transfer of organisms during transit and travel. These regulations establish protocols to monitor and manage the movement of goods and people, especially at border checkpoints with high levels of passenger and vehicle traffic. This helps reduce the chances of vectors organisms unintentionally entering as “stowaways”. Additionally, trade regulations can enforce the execution of educational initiatives aimed at raising public awareness about the risks associated with transporting foreign arthropod species, and the necessary preventive actions that should be implemented [56].

**Policy and legal framework for migrants’ healthcare provision**: for instance, the EU accession process and the requirement for harmonization between national and EU legislation were the driving forces behind recent modifications to Croatia’s legal framework regarding the provision of healthcare for migrants. The purpose of these modifications was to bring the Asylum Act into compliance with Croatia’s Law on Compulsory Health Insurance and Health care for immigrants, which governs immigrants’ mandatory health insurance as well as their access to healthcare. The changes imposed restrictions on the supply of healthcare services to immigrants, such as the mandate that foreign nationals holding both temporary and permanent residency permits be covered by the mandatory state-run health insurance program. Additionally, the changes specified that migrants not entitled to compulsory health insurance must pay a monthly fee to the Croatian Health Insurance Fund. Irregular migrants are entitled to emergency care, defined as necessary diagnostic and therapeutic measures to remove immediate threats to life and health [45].

**The “Health, Border and Mobility Management Framework”** developed by the IOM aims to provide communities and governments with the capacity to prevent, identify, and manage health risks to the public across the mobility spectrum. In the context of global migration, it emphasizes the importance of integrating health, border, and mobility management, especially in response to communicable diseases such as cholera, COVID-19, EVD, and yellow fever. It highlights the need for multisectoral coordination to effectively address public health threats posed by population movements. The framework stresses the importance of evidence-based strategies, strengthening health systems, capacity-building at POEs, community engagement, inclusive policy frameworks, and strong partnerships to tackle public health challenges in the context of migration. Furthermore, the framework underscores the significance of conducting PMM exercises to gather essential quantitative and qualitative data on mobility patterns and vulnerabilities at national and subnational levels, as well as at POEs and informal border crossings. These exercises help identify priority communities and locations that may be at risk of infectious disease outbreaks and other health hazards, informing public health preparedness and response efforts. Additionally, it aims to promote gender equality by reducing gender disparities. The framework supported malaria elimination efforts in Paraguay, which was certified as malaria-free by the WHO in 2018 [57].

The **Accountability to Affected Population (AAP) Framework** is a proactive commitment made by humanitarian actors to exercise their authority responsibly by being accountable for supporting the people they seek to assist. The AAP Framework was created by IOM following the IASC’s commitments. Its main goal is to ensure that programming is centered around the affected people. The main areas of focus for AAP commitments are (a) leadership; (b) transparency and information sharing; (c) involvement; (d) channels for complaints and criticism; and (e) partner collaboration. It reinforces a zero-tolerance policy against sexual exploitation and abuse (SEA) and other forms of misconduct [57].

##### Schengen Agreement and Early Warning and Response System

The Schengen Agreement is a cooperation instrument between member states that covers issues of border control, data protection, and visa and consular cooperation. In terms of health-related rules, the regulations aim to prevent any potential threats to the public health of member states. Member states have the authority to deny entry to individuals who may pose a risk to public health [13].

International and European instruments have established fundamental principles and criteria to ensure the health and well-being of individuals, particularly migrants in reception and detention facilities. According to these standards, the physical and mental well-being, safety, and access to social services of all individuals in detention, including those under immigration control measures involving detention, must meet basic minimum standards. The European Prison Rules and the U.N. Standard Minimum Rules for the Treatment of Prisoners are legally binding manuals that outline how to uphold regional and global commitments to protect the human rights of those detained in all types of detention facilities, including general material conditions and basic access to services [13].

#### 3.2.8. Services and Assistance for At-Risk Groups

Pre-existing vulnerabilities, discrimination, and violence, which may overlap with other characteristics such as gender, age, disability, nationality, and/or ethnic origin, can be worsened by public health emergencies [57]. Protecting migrants and other vulnerable populations is essential in any intervention. Those in vulnerable situations or in need of protection, such as women at risk or survivors of gender-based violence (GBV), people with disabilities, unaccompanied and separated children, elderly individuals, stranded migrants, displaced populations, and affected communities, are considered part of the at-risk population.

Protection mechanisms and responses during public health and other emergencies involve providing urgent protection services or referrals to appropriate services for those in need. Examples of protection services include emergency shelter, alternative care, family tracing and reunification, and access to additional health services, such as case management, livelihood assistance, and Mental Health Psychosocial Assistance (MHPSS) [57].

To establish an integrated system for human rights protection measures, it is crucial to establish appropriate collaboration procedures among relevant agencies and organizations both nationally and internationally [12].

## 4. Discussion

To our knowledge, this is the first scoping review conducted on the main activities that should be included for effective health management at border crossings. The study included 45 papers from which eight main themes were identified.

Of the themes, “IPC measures” and “Collaboration, Coordination, and Partnership” had the highest frequency among the studies and reports published (17% each). The second highest frequency was for “Research and Data Sharing” (15%), followed by “Building Border Health Capacity” and “Planning” (13.5% each), “Communication” (13%), “Legislation and Framework” (7%), and finally, “Services and Assistance for At-Risk Groups” (4%).

In line with existing literature, IPC measures are crucial to implement at border crossings, especially during mass movements due to wars and natural disasters when prevention and control programs may deteriorate. To prevent cross-border transmission of infections, it is critical to ensure that minimum requirements are implemented at both the facility and national levels. This includes creating screening and referral mechanisms for infected cases as well as effective case management to prevent the spread of infection without interfering with international trade and travel. Real-time surveillance plays a crucial role in detecting and responding to outbreaks in humanitarian emergencies, so surveillance and the EWARS need to be strengthened to ensure countries are prepared for future public health threats. Additionally, mass vaccination campaigns are needed for VPDs [11,12,23,24].

Research has shown that collaboration, coordination, and partnerships are highly recommended at national and international levels to strengthen global health security. Cross-border collaboration to enhance the information-sharing and coordination capacity of VPDs across borders, especially between developed and developing countries, should be promoted. Establishing platforms for information exchange and coordination helps in responding effectively to health emergencies through collaboration between the WHO, other UN agencies, NGOs, and border officials, building on past experiences with cross-border activities for other communicable diseases [27,41].

Research and data sharing are strongly advised to respond to health emergencies. With accurate information, countries can allocate resources effectively and collaborate with partners to address gaps. Real-time access to accurate information helps decision-makers make effective and informed decisions, resulting in the saving of lives and mitigation of the impact of health crises. This can be seen in the studies conducted for malaria control [37].

Building land border capacities is crucial for enhancing national security and ensuring the smooth movement of people and goods. Investing in the training of border personnel ensures they have the necessary skills to effectively manage various challenges. Strengthening border infrastructure, including checkpoints, WASH systems, and surveillance systems, helps to detect and prevent health threats and illegal activities, such as smuggling, trafficking, and unauthorized crossings. Increasing financial resources at land borders is essential for improving economic stability, efficiency, and security. Adequate funding enables the implementation of updated border infrastructure, checkpoints, enhanced transportation routes, and innovative surveillance technology. This investment prepares border control agencies to handle difficulties related to immigration, illegal trafficking, security, and health risks. Additionally, improved funding allows border officials to receive better assistance and training, enhancing their capacity to manage challenging circumstances. Strong financial resources also support trade and travel, reducing delays and facilitating seamless cross-border exchanges. This encourages commerce, boosts economic growth, and strengthens global trust and collaboration. According to the literature, countries should assess their current border capacities, identify gaps, and develop strategies to enhance their capacities [4,42,54]

Furthermore, planning and developing SOPs for health management at land borders is critically important for preventing and controlling the spread of infectious diseases. Effective health management plans provide SOPs for screening, diagnosing, and case management of travelers who may carry infectious diseases, reducing the risk of cross-border transmission. SOPs ensure that border facilities are well equipped, surveillance and emergency plans are in place, and border personnel are trained to respond to health emergencies at borders. Health management planning at land borders also facilitates the implementation of vaccination programs and health education initiatives, raising awareness about disease prevention among travelers. This proactive approach helps maintain public health security and minimizes disruptions to travel and trade [47,49,50].

Moreover, for effective health border management at land borders, having an efficient communication strategy is crucial. This ensures prompt, well-coordinated, and effective responses to health hazards. Clear and consistent communication between border authorities, health officials, and other relevant parties allows for the exchange of vital information in real time, including updates on disease outbreaks, health screening guidelines, and emergency measures [10,30,53].

Effective communication techniques ensure that border staff members are well informed and capable of carrying out health procedures accurately and efficiently, thereby reducing the risk of disease transmission. They also foster transparency and trust among travelers, who need to be informed about any potential risks and health requirements associated with cross-border travel. Moreover, good communication facilitates cross-border cooperation by enabling neighboring countries and international health organizations to exchange knowledge and best practices. Cooperation is essential to effectively handle health crises and ensure that actions taken on one side of the border are complemented by those on the other.

Strong communication channels also aid in the mobilization of resources and the coordination of logistics, such as the distribution of medical supplies and deployment of medical personnel to border areas. This is particularly critical during emergencies when timely and well-coordinated responses are vital.

In addition, enforcing laws and establishing a strong framework for health border management is necessary to ensure organized, reliable, and efficient responses to health risks at land borders. These legal frameworks provide clear guidelines and protocols for border health management, serving as a basis for coordinated action among various stakeholders, including border authorities, health agencies, and international partners. They also ensure that health measures at borders are standardized and uniformly enforced, helping to prevent the spread of infectious diseases and other health risks. By providing the legal authority for implementing necessary actions, such as health screenings, quarantines, and travel restrictions, these frameworks ensure that these measures are applied effectively. Governments can allocate funding and resources to border health management more effectively when there is a clear legal framework in place. This includes the allocation of medical supplies, training for border workers, and the construction of necessary infrastructure for emergency response and health screenings. Harmonized legal frameworks also promote international collaboration and information exchange, ensuring that health interventions are coordinated across borders to efficiently address transboundary health concerns [45,56].

Developing strategies for services and assistance for at-risk groups at land borders is crucial for effective health border management. Implementing targeted strategies ensures that vulnerable populations receive appropriate care and support, reducing the risk of disease transmission and promoting overall public health. These strategies may include vaccination against preventable diseases, health screening, health education, awareness campaigns on human trafficking and reporting channels, psychosocial support, and providing legal and social assistance [57].

These findings can provide practical applications and policy recommendations that will enhance the effectiveness of health management at ground border crossings. IPC protocols should be implemented for both developed and developing countries, considering resource constraints and cultural differences. Training programs should be held for border personnel along with the allocation of dedicated funds for IPC efforts. Establishing international health networks and formal agreements for data and resource sharing, communication, and collaboration during emergencies is essential. Investments in real-time data platforms and regional data-sharing agreements are recommended to manage outbreaks and ensure transparency. Border health infrastructure should be adaptable to changing traffic and emergencies, with regular assessments and support from international donors. Legal frameworks should harmonize health-related legislation across countries, defining roles for stakeholders and ensuring compliance with the IHR. Special attention is needed for at-risk populations, with tailored interventions like vaccination and screenings integrated into broader public health strategies to ensure sustainability.

More research is needed especially in the context of cross-border disease surveillance and health data sharing. This includes integrating technology as well as capacity building and workforce training in border health management. The impact of mobility patterns on health outcomes, public health preparedness, and migration, as well as health access for migrants and refugees, is also important. By conducting research in these areas, policymakers and health organizations can improve preparedness and responses to health issues at border crossings, ultimately enhancing both mobility management and public health outcomes.

## 5. Conclusions

This scoping review emphasizes the multifaceted nature of health security challenges at border crossings. By systematically analyzing studies published between January 2005 and December 2023, we have identified eight critical themes essential for effective health management at border crossings. These themes are interconnected and collectively crucial for improving emergency preparedness and response. Addressing these areas comprehensively can encourage policymakers to establish policies, recommendations, and frameworks that greatly enhance health security at border crossings, ensuring a more coordinated and effective response to health threats and humanitarian emergencies such as forced migration. Countries must allocate funds to build border health capacities, set IPC measures, and integrate technology, data sharing, and communication strategies for effective health management.

Evidence-based research and frequent on-site assessments are necessary to address the gaps at border crossings and implement frameworks that uphold global health security.

## 6. Limitations

The main limitation of this study is that 55% of the included papers are from grey literature, as ground crossing borders are understudied. Despite this high percentage, these papers play a crucial role in shaping real-world practices and provide a comprehensive understanding of health border management at ground crossing borders, compared to when limiting the scope to academic research alone. Another limitation is that only English publications were included in the literature search, which introduces a risk of language bias. Additionally, excluding non-open access papers could lead to selection bias.

## Figures and Tables

**Table 1:** Themes extracted, their frequency, and percentage.

Theme:	Frequency	Percentage
I. Infection Prevention and Control Measures	20	17
II. Collaboration, Coordination, and Partnership	20	17
III. Research and Data Sharing	18	15
IV. Building Border Health Capacity	16	13.5
V. Planning	16	13.5
VI. Communication	15	13
VII. Legislations and Frameworks	8	7
VIII. Services and Assistance for At-Risk Groups	5	4
Total	118	100

Frequency: No. of occurrences of theme in the papers. More than one theme can be found in a paper. Percentage: (Frequency/Total Frequency) × 100.

## Supplementary Materials

The following supporting information can be downloaded at: https://www.mdpi.com/article/10.3390/healthcare12191968/s1, Table S1: Characteristics of the Papers included in the Scoping Review.
