# Supplementary material for: Strengthening Health Security at Ground Border Crossings: Key Components for Improved Emergency Preparedness and Response—A Scoping Review
Source: Healthcare (Basel). 2024 Oct 2;12(19):1968. doi: 10.3390/healthcare12191968 (PMC11476094; doi:10.3390/healthcare12191968)
Supplement: Supplementary file 1 [file healthcare-12-01968-s001.zip › healthcare-3185359-supplementary.pdf]

Supplementary Table 1: Characteristics of the Papers included in the Scoping Review.

SERIAL NO.	LOCATION	PUBLICATION TYPE	THEME	PUBLISHER	YEAR	PUBLICATION
1	European Economic Area, Hungary, Poland, Romania, Slovakia	Manual/ Training guide	• BUILD BORDER HEALTH CAPACITY	IOM	2010	Training modules on Migration and Health for Border Officials
2	European Economic Area, Hungary, Poland, Romania, Slovakia, South-Eastern Europe Eastern Europe and Central Asia, Belarus, Ukraine	TECHNICAL REPORT	• LEGISLATIONS and FRAMEWORK<br>• BUILD BORDER HEALTH CAPACITY<br>• IPC MEASURES<br>• SERVICE and ASSISTANCE FOR AT RISK GROUP	IOM	2010	Increasing public health safety alongside the New Eastern European Border: An overview of findings from the situational analysis
3	European Economic Area, Hungary, Poland, Romania, Slovakia, Global, South-Eastern Europe Eastern Europe and Central Asia, Belarus, Ukraine	Manual/ Training guide	• SERVICE and ASSISTANCE FOR AT RISK GROUP<br>• PLANNING	IOM	2010	Guidelines for border management and detention procedures involving migrants: A public health perspective
4	European Economic Area, Hungary, Poland, Slovakia	SCIENTIFIC REPORT(Journal)	• RESEARCH and DATA SHARING	IOM	2011	Health, migration and border management: analysis and capacity-building at Europe's borders
5	Italy	SITUATION REPORT	• COORDINATION and PARTNERSHIP<br>• BUILD BORDER HEALTH CAPACITY	IOM	2015	Italy Health Situation at EU's Southern Borders – Assessment Report

Supplementary Table 1: Characteristics of the Papers included in the Scoping Review.

SERIAL NO.	LOCATION	PUBLICATION TYPE	THEME	PUBLISHER	YEAR	PUBLICATION
			• SERVICE and ASSISTANCE FOR AT RISK GROUP<br>• RESEARCH and DATA SHARING<br>• PLANNING<br>• LEGISLATIONS and FRAMEWORK			
6	Global	TECHNICAL DOCUMENT-ADVISING PRINCIPLES	• COORDINATION and PARTNERSHIP<br>• COMMUNICATION	WHO	2018	Coordination of public health surveillance between points of entry and the national public health surveillance system
7	Global	TECHNICAL DOCUMENT HANDBOOK	• PLANNING<br>• IPC MEASURES	WHO	2016	Vector surveillance and control at ports, airports, and ground crossings
8	Global	TECHNICAL DOCUMENT GUIDELINE	• PLANNING<br>• COMMUNICATION<br>• RESEARCH and DATA SHARING<br>• COORDINATION and PARTNERSHIP<br>• BUILD BORDER HEALTH CAPACITY<br>• IPC MEASURES	WHO	2020	International health regulations (2005): assessment tool for core capacity requirements at designated airports, ports and ground crossings
9	Global	REPORT	• IPC MEASURES	WHO	2014	Early detection, assessment and response to acute public health events: implementation of early warning and response with a focus on event-based surveillance
10	Global	TECHNICAL DOCUMENT GUIDELINE	• PLANNING	WHO	2012	International health regulations (2005) : a guide for public health emergency

Supplementary Table 1: Characteristics of the Papers included in the Scoping Review.

SERIAL NO.	LOCATION	PUBLICATION TYPE	THEME	PUBLISHER	YEAR	PUBLICATION
						contingency planning at designated points of entry
11	Global	TECHNICAL DOCUMENT HANDBOOK	• BUILD BORDER HEALTH CAPACITY	WHO	2020	Handbook for public health capacity-building at ground crossings and cross-border collaboration
12	Global	REPORT	• RESEARCH and DATA SHARING<br>• IPC MEASURES	WHO	2010	Public health measures taken at international borders during early stages of pandemic influenza A (H1N1) 2009
13	Global	TECHNICAL DOCUMENT GUIDELINE	• PLANNING<br>• IPC MEASURES	WHO	2014	Exit screening at airports, ports and land crossings: Interim guidance for Ebola virus disease
14	Global	TECHNICAL DOCUMENT GUIDELINE	• PLANNING<br>• IPC MEASURES	WHO	2014.	Technical note for Ebola preparedness planning for entry screening at airports, ports and land crossings
15	Global	TECHNICAL DOCUMENT GUIDELINE	• PLANNING<br>• IPC MEASURES	WHO	2014	Ebola Event Management at Points of Entry
16	Global	TECHNICAL DOCUMENT GUIDELINE	• RESEARCH and DATA SHARING<br>• BUILD BORDER HEALTH CAPACITY<br>• PLANNING	WHO	2017	Joint external evaluation tool: International Health Regulations (2005) - second edition
17	Global	TECHNICAL REPORT	• IPC MEASURES<br>• COORDINATION and PARTNERSHIP	IOM	2022	COVID-19 and the State of Global Mobility in 2021
18	Global	Brochure/ Factsheet/ Infosheet	• RESEARCH and DATA SHARING<br>• IPC MEASURES	IOM	2021	Population Mobility Mapping

Supplementary Table 1: Characteristics of the Papers included in the Scoping Review.

SERIAL NO.	LOCATION	PUBLICATION TYPE	THEME	PUBLISHER	YEAR	PUBLICATION
19	Nepal	TECHNICAL REPORT	• BUILD BORDER HEALTH CAPACITY<br>• COORDINATION and PARTNERSHIP<br>• PLANNING<br>• LEGISLATIONS and FRAMEWORK<br>• IPC MEASURES<br>• SERVICE and ASSISTANCE FOR AT RISK GROUP<br>• RESEARCH and DATA SHARING<br>• COMMUNICATION	IOM	2021	Assessing the Ground Crossing Points of Nepal and Their Compliance with the International Health Regulations (2005) to Prepare and Inform the Public Health Response to COVID-19
20	Global	TECHNICAL REPORT	• RESEARCH and DATA SHARING<br>• BUILD BORDER HEALTH CAPACITY<br>• LEGISLATIONS and FRAMEWORK<br>• COMMUNICATION<br>• COORDINATION and PARTNERSHIP	IOM	2021	Health, Border and Mobility Management Framework: A Framework to Empower Governments and Communities to Prevent, Detect and Respond to Public Health Threats along the Mobility Continuum
21	Global	Brochure/ Factsheet/ Infosheet	• COMMUNICATION<br>• BUILD BORDER HEALTH CAPACITY	IOM	2020	Training Curriculum for Border Officials on COVID-19 Response at Points of Entry
22	Global	Brochure/ Factsheet/ Infosheet	• COMMUNICATION<br>• PLANNING	IOM	2020	IOM Tools for Border Officials and Migrants for COVID-19 Response
23	Global	Brochure/ Factsheet/ Infosheet	• BUILD BORDER HEALTH CAPACITY<br>• COMMUNICATION<br>• LEGISLATIONS and FRAMEWORK<br>• COORDINATION and PARTNERSHIP	IOM	2020	COVID-19 Immigration and Border Management Response

Supplementary Table 1: Characteristics of the Papers included in the Scoping Review.

SERIAL NO.	LOCATION	PUBLICATION TYPE	THEME	PUBLISHER	YEAR	PUBLICATION
24	Southern Africa	SITUATION REPORT	• COORDINATION and PARTNERSHIP<br>• IPC MEASURES<br>• RESEARCH and DATA SHARING<br>• COMMUNICATION	IOM	2018	IOM DR Congo Ebola Response Bulletin ǀ 19 June 2018 ǀ Situation Report 3
25	European Economic Area, Croatia	TECHNICAL REPORT	• LEGISLATIONS and FRAMEWORK<br>• BUILD BORDER HEALTH CAPACITY<br>• RESEARCH and DATA SHARING<br>• COORDINATION and PARTNERSHIP<br>• SERVICE and ASSISTANCE FOR AT RISK GROUP<br>• IPC MEASURES	IOM	2015	Croatia Health Situation at EU's Southern Borders - Assessment Report
26	Global	ASSESSMENT TOOL	• BUILD BORDER HEALTH CAPACITY	BMC PUBLIC HEALTH	2010	Global health security and the International Health Regulations
27	CARRIBBEAN	COMMENTARY	• COORDINATION and PARTNERSHIP	HEALTH SYSTEMS and REFORM	2018	Fighting Health Security Threats Requires a Cross-Border Approach
28	WEST AFRICA	CASE REPORT	• COMMUNICATION	BMC INFECTIOUS DISEASES	2019	Investigation of a cross-border case of Lassa fever in West Africa
29	AFRICA	RESEARCH ARTICLE	• PLANNING<br>• BUILD BORDER HEALTH CAPACITY<br>• COORDINATION and PARTNERSHIP	EMERGING INFECTIOUS DISEASES	2017	Responding to Communicable Diseases in Internationally Mobile Populations at Points of Entry and along Porous Borders, Nigeria, Benin, and Togo
30	WEST AFRICA	REPORT	• COMMUNICATION<br>• COORDINATION and PARTNERSHIP<br>• IPC MEASURES	MMWR-MORBIDITY AND MORTALITY WEEKLY REPORT	2016	Travel and Border Health Measures to Prevent the International Spread of Ebola

Supplementary Table 1: Characteristics of the Papers included in the Scoping Review.

SERIAL NO.	LOCATION	PUBLICATION TYPE	THEME	PUBLISHER	YEAR	PUBLICATION
31	UGANDA	RESEARCH ARTICLE	• PLANNING	HEALTH SECURITY	2020	Ebola Virus Disease Preparedness Assessment and Risk Mapping in Uganda, August-September 2018
32	Global	CHAPTER	• COORDINATION and PARTNERSHIP<br>• COMMUNICATION<br>• RESEARCH and DATA SHARING<br>• BUILD BORDER HEALTH CAPACITY	URBAN DISASTER RESILIENCE AND SECURITY: ADDRESSING RISKS IN SOCIETIES	2018	Challenges in Establishing Cross-Border Resilience
33	GUINEA	RESEARCH ARTICLE	• RESEARCH and DATA SHARING<br>• COORDINATION and PARTNERSHIP<br>• IPC MEASURES<br>• COMMUNICATION	FRONTIERS IN PUBLIC HEALTH	2022	Lessons learned for surveillance system strengthening through capacity building and partnership engagement in post-Ebola Guinea, 2015-2019
34	AFRICA	RESEARCH ARTICLE	• RESEARCH and DATA SHARING	HEALTH SECURITY	2021	Lessons Learned From a Large Cross-Border Field Simulation Exercise to Strengthen Emergency Preparedness in East Africa, 2019
35	UGANDA	RESEARCH ARTICLE	• IPC MEASURES<br>• RESEARCH and DATA SHARING	ZOONOSES AND PUBLIC HEALTH	2021	Preventing the cross-border spread of zoonotic diseases: Multisectoral community engagement to characterize animal mobility-Uganda, 2020
36	NIGERIA, TOGO, BENIN	RESEARCH ARTICLE	• COMMUNICATION<br>• COORDINATION and PARTNERSHIP	HEALTH SECURITY	2020	Improving Cross-Border Preparedness and Response: Lessons Learned from 3

Supplementary Table 1: Characteristics of the Papers included in the Scoping Review.

SERIAL NO.	LOCATION	PUBLICATION TYPE	THEME	PUBLISHER	YEAR	PUBLICATION
			• RESEARCH and DATA SHARING			Lassa Fever Outbreaks Across Benin, Nigeria, and Togo, 2017-2019
37	UGANDA	RESEARCH ARTICLE	• COMMUNICATION	FRONTIERS IN PUBLIC HEALTH	2023	Risk perception of Ebola virus disease and COVID-19 among transport drivers living in Ugandan border districts
38	AFRICA	RESEARCH ARTICLE	• IPC MEASURES<br>• RESEARCH and DATA SHARING	MALARIA JOURNAL	2014	Quantifying cross-border movements and migrations for guiding the strategic planning of malaria control and elimination
39	DEVELOPING COUNTRIES	RESEARCH ARTICLE	• COORDINATION and PARTNERSHIP<br>• BUILD BORDER HEALTH CAPACITY<br>• IPC MEASURES<br>• PLANNING	PAN AFRICAN MEDICAL JOURNAL	2021	COVID-19 pandemic in Economic Community of West African States (ECOWAS) region: implication for capacity strengthening at Point of Entry
40	UGANDA	RESEARCH ARTICLE	• RESEARCH and DATA SHARING<br>• PLANNING<br>• BUILD BORDER HEALTH CAPACITY<br>• COORDINATION and PARTNERSHIP<br>• IPC MEASURES	GLOBALIZATION AND HEALTH	2020	Uganda's experience in Ebola virus disease outbreak preparedness, 2018-2019
41	ASIA	RESEARCH ARTICLE	• COMMUNICATION<br>• COORDINATION and PARTNERSHIP	PROGRESS IN DISASTER SCIENCE	2020	COVID-19 and ASEAN responses: Comparative policy analysis
42	POLAND	BRIEF REPORT	• COORDINATION and PARTNERSHIP	SUSTAINABILITY	2019	The Territorial Defence Force in Disaster Response in Poland: Civil-Military Collaboration

Supplementary Table 1: Characteristics of the Papers included in the Scoping Review.

SERIAL NO.	LOCATION	PUBLICATION TYPE	THEME	PUBLISHER	YEAR	PUBLICATION
						during a State of Emergency
43	ASIA	REVIEW	• LEGISLATIONS and FRAMEWORK	Roczniki Panstwowego Zakladu Higieny	2019	Development of land transport connections between Asia and Europe and their possible impact on vector introductions into European Countries.
44	US	RESEARCH ARTICLE	• LEGISLATIONS and FRAMEWORK<br>• RESEARCH and DATA SHARING<br>• COORDINATION and PARTNERSHIP<br>• IPC MEASURES	Public health reports (Washington, D.C. : 1974)	2009	A new paradigm for quarantine and public health activities at land borders: opportunities and challenges.
45	Global	GUIDELINES	• PLANNING	Romanian Journal of Infectious Diseases	2010	INTERNATIONAL HEALTH REGULATIONS 2005 – TECHNICAL APPROACH OF THE SURVEILLANCE AND CONTROL OF INTERNATIONAL DEVELOPMENTS OF ACUTE INFECTIOUS DISEASES
